# Impact of mindfulness practices to improve quality of life and mental health of persons diagnosed with breast cancer: a systematic review

**DOI:** 10.3389/fpsyg.2025.1641751

**Published:** 2025-11-20

**Authors:** Rithika Menon, T. S. Saranya

**Affiliations:** 1 School of Liberal Studies, CMR University, Bengaluru, India; 2 Clinical Psychology Department, Amity Institute of Behavioural Health and Allied Sciences, Amity University Bengaluru, Bengaluru, India

**Keywords:** breast cancer, mindfulness-based interventions, psychological distress, quality of life, coping, depression, anxiety

## Abstract

**Introduction:**

Cancer remains one of the leading causes of mortality worldwide and has a profound impact on an individual’s psychological well-being. Individuals diagnosed with breast cancer often experience anxiety, depression, and reduced quality of life (QoL) due to the physical and emotional burden of the disease. Mindfulness-based interventions have emerged as effective strategies to alleviate psychological distress and enhance overall well-being. This systematic review aims to examine the impact of various mindfulness practices on the mental health and quality of life of individuals diagnosed with breast cancer.

**Methods:**

A comprehensive literature search was conducted following the PRISMA guidelines across multiple electronic databases, including Google Scholar, Web of Science, PubMed, and Scopus. Studies focusing on mindfulness interventions specifically designed for individuals diagnosed with breast cancer were included. Studies addressing other types of cancer or cancer survivors were excluded. The selected studies were screened through title, abstract, and full-text reviews based on predefined inclusion and exclusion criteria.

**Results:**

A total of 20 studies met the inclusion criteria and were analyzed. The majority of the studies employed Mindfulness-Based Stress Reduction (MBSR) as the primary intervention technique. Although variations existed in the number and duration of sessions across studies, the overall findings consistently demonstrated significant improvements in participants’ mental health outcomes—including reductions in anxiety, depression, and emotional distress—and enhanced quality of life following mindfulness interventions.

**Discussion:**

This systematic review provides strong evidence supporting the effectiveness of mindfulness practices, particularly MBSR, in improving psychological well-being and QoL among individuals diagnosed with breast cancer. The findings emphasize the importance of integrating mindfulness-based interventions into cancer care programs to address the psychological challenges associated with diagnosis and treatment. Future research should aim to standardize intervention protocols and explore long-term effects on mental health and quality of life.

## 1 Introduction

Cancer is considered one of the top five chronic diseases prevalent in India and can pose a negative impact on an individual’s quality of life (QoL). It is considered one of the leading causes of death, with approximately 1.16 million new cases being diagnosed per year. In India, the most common types of cancers being diagnosed are breast, lung, and cervical cancer (Arora, 2023). In a study conducted in India, researchers assessed breast cancer survival after 5 years of diagnosis across 11 geographic areas under the National cancer registry program and found it to be 66.4% (Sathishkumar et al., 2024).

Due to the stigma surrounding cancer, the term is often considered emotionally charged due to its serious nature. Research shows that the word “cancer” tends to trigger heightened emotions such as fear, anxiety, and even alarming thoughts, regardless of an individual’s true health status (National Cancer Institute, 2025). This reaction is linked to the strong associations cancer has with suffering, death, and uncertainty. In many societies, cancer is closely tied to the idea of dying, which amplifies its mental impact. As a result, even a simple mention of the term can lead to feelings of fear and unease, demonstrating the significant psychological burden linked to the illness (Vrinten et al., 2017). Cancer poses a wide range of emotional, physical, and financial challenges among patients, and if left untreated, can result in poor QoL and overall well-being. Some of the commonly seen emotional symptoms in cancer patients due to diagnosis and treatment include anxiety, depression, fear, anger, stress, fatigue, isolation, and concerns about body image, mortality, and finance (Choudhary, 2024). Globally, breast cancer remains the most commonly diagnosed cancer among women, accounting for approximately 2.3 million new cases and 685,000 deaths annually (World Health Organization, 2023). The disease poses a significant public health challenge in both high-income and low- and middle-income countries (LMICs). India, as one of the largest LMICs, exemplifies this growing burden, with breast cancer representing nearly 14% of all female cancer cases and a rising incidence in both urban and rural areas (Sathishkumar et al., 2022). The inclusion of Indian data serves as an illustrative example of LMIC trends, where limited awareness, delayed diagnosis, and disparities in access to psychosocial care exacerbate mental health challenges among patients. Presenting this context emphasizes the global significance of addressing both the physical and psychological dimensions of breast cancer care. Studies have found that in India, the prevalence of depression among cancer patients is about 27%, with an approximate 10% suffering from anxiety (Chadha, 2024). Vaseel and Uvais’ (2024) study stated that the prevalence of depression in persons diagnosed with breast cancer varies across studies, ranging from 21.5–83.5%. Another study showed results within a similar range for depression (37.9%) and anxiety (33.6%) (Dadheech et al., 2023). Therefore, persons diagnosed with breast cancer are at high risk of developing psychiatric comorbidities and must be regularly monitored by Mental Health Professionals. It is also suggested to develop an effective intervention to help overcome these increasing comorbidities among persons diagnosed with breast cancer (Mir et al., 2023).

Patients diagnosed with breast cancer frequently endure considerable psychological turmoil, which encompasses anxiety, depression, and apprehension regarding recurrence. This distress can significantly diminish their QoL both during and after medical treatment (National Cancer Institute, 2022). Mindfulness practices, which involve a deliberate and non-judgmental awareness of current experiences, have been identified as effective interventions to reduce these problems (Lengacher et al., 2016). Such practices generally encompass meditation, body scanning, mindful movement, and breathing exercises that support patients in cultivating psychological adaptability and emotional regulation capabilities (Piet et al., 2012). Initial studies illustrated the efficacy of Mindfulness-Based Stress Reduction (MBSR) in addressing chronic pain and anxiety (Kabat-Zinn, 1982), with later modifications specifically devised for cancer patients (Carlson et al., 2013). Recent systematic reviews have suggested that mindfulness interventions can alleviate psychological distress, enhance emotional health, and improve overall QoL for persons diagnosed with breast cancer across different phases of diagnosis, treatment, and survivorship (Zimmermann et al., 2018; Haller et al., 2017; Zhang et al., 2018). These outcomes have led to a growing incorporation of mindfulness practices into holistic cancer care programs globally (Greenlee et al., 2017), although uncertainties persist regarding the most effective timing, format, and duration of interventions for diverse patient populations.

Even though various psychological and mindfulness-based therapies are being studied for persons diagnosed with breast cancer, there is still scattered data in various different methods and outcomes. The results of mindfulness interventions during active therapy are not specifically examined in a comprehensive review of prior research. This systematic review aimed to critically assess the existing literature and provide additional insight into the impact of Mindfulness practices on QoL and mental health among persons diagnosed with breast cancer.

## 2 Methodology

### 2.1 Search strategy

A systematic review was conducted by following the PRISMA (Preferred Reporting Items for Systematic Reviews and Meta-Analyses) guidelines. Potential studies were identified by searching Databases including Google Scholar, Web of Science, PubMed and Scopus using keywords “breast cancer,” “mindfulness-based interventions,” “psychological distress,” “quality of life,” “coping,” “depression” and “anxiety.” The study population included women who had been diagnosed with breast cancer and were undergoing treatment. There were no criteria for the stage of breast cancer in the studies. The primary outcome of this review was the mental health and QoL of women with breast cancer after receiving mindfulness interventions.

### 2.2 Inclusion criteria

Inclusion criteria were developed to ensure that the studies were relevant to the research objectives. Studies that fulfilled the following criteria were included:

1. Studies that were published between 2015 and 2025 timeframe.
2. Population: Adults aged 18 years and older who have been diagnosed with breast cancer, whether they belong solely to this group or are part of a broader cancer population, were included as long as results pertaining to breast cancer patients could be identified or extracted. The study encompassed both individuals currently receiving treatment for cancer and those who are cancer survivors or living with cancer.
3. Intervention: Mindfulness-based interventions (MBIs) that specifically target the enhancement of psychological health or QoL were considered. Eligible interventions consisted of, but were not limited to, MBSR, Mindfulness-Based Cognitive Therapy (MBCT), Mindfulness-Based Art Therapy (MBAT), Mindfulness-Based Music Therapy (MBMT), as well as other organized mindfulness initiatives. Acceptable delivery formats included both face-to-face and technology-facilitated methods (online or hybrid).
4. Study Design: The studies must include randomized controlled trials (RCTs), quasi-experimental studies, controlled clinical trials, pilot studies, and pre-post intervention assessments that present quantitative results. Studies utilizing mixed-methods were eligible for inclusion if they provided quantitative data.
5. Language: There were no restrictions regarding language. Publications in languages other than English were translated and evaluated for inclusion.
6. Outcomes: It was necessary for studies to evaluate at least one quantitative psychological or quality-of-life outcome through validated instruments (for instance, anxiety, depression, stress, coping, or overall QoL). Additionally, secondary psychosocial outcomes such as fatigue, emotional regulation, or general well-being were also eligible for consideration.

### 2.3 Exclusion criteria

Certain studies were excluded to maintain the quality of the research. Studies with the following criteria were excluded:

1. Did not consider individuals diagnosed with breast cancer or those who have survived it (Individuals Living with Cancer) as a portion of the study population.
2. Did not execute a mindfulness-oriented intervention (such as Mindfulness-Based Stress Reduction, Mindfulness-Based Cognitive Therapy, Mindfulness-Based Art Therapy, yoga, Mindfulness-Based Movement Therapy, or any other organized mindfulness practices).
3. Did not evaluate outcomes associated with mental health (such as depression, anxiety, psychological distress) or QoL employing validated assessment instruments.
4. Included review articles, meta-analyses, editorials, commentaries, letters, conference abstracts lacking comprehensive text data, or dissertations.
5. Included case studies or individual case reports that do not yield generalizable conclusions.
6. Concentrated solely on non-human subjects or experimental models situated in a laboratory environment.
7. Lacked adequate data necessary for extraction and thorough analysis (for instance, absent outcome measures, incomplete reporting).

### 2.4 Results of search process

**Figure 1:**
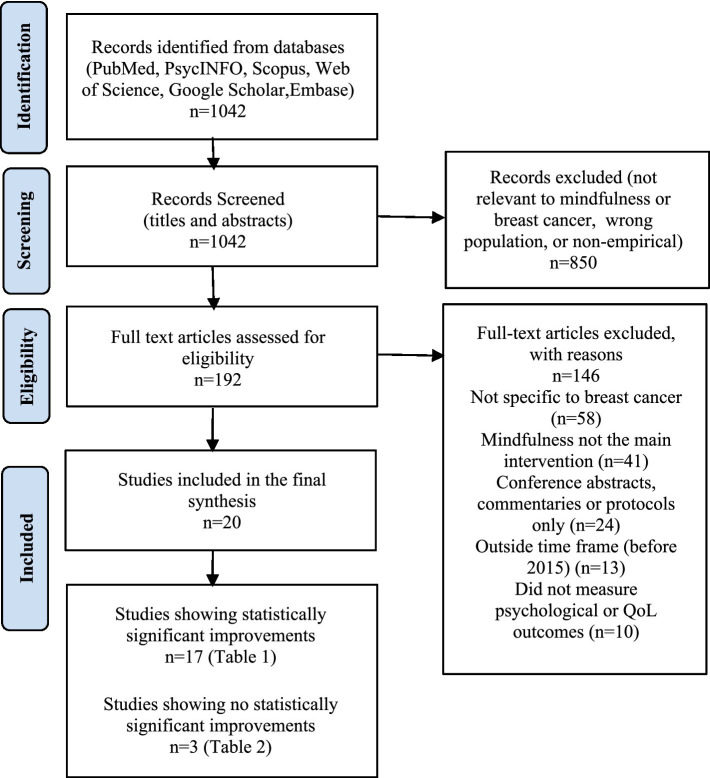
Flowchart of study selection according to PRISMA guidelines.

A systematic search across four databases initially identified 24,513 records. After applying inclusion and exclusion criteria, duplicates, reviews, non–peer-reviewed studies, studies not focused on MBIs, and studies not addressing mental health or QoL in breast cancer patients were excluded. Following this rigorous screening process, 20 studies were retained for final synthesis (see Figure 1).

## 3 Results

**Figure 2:**
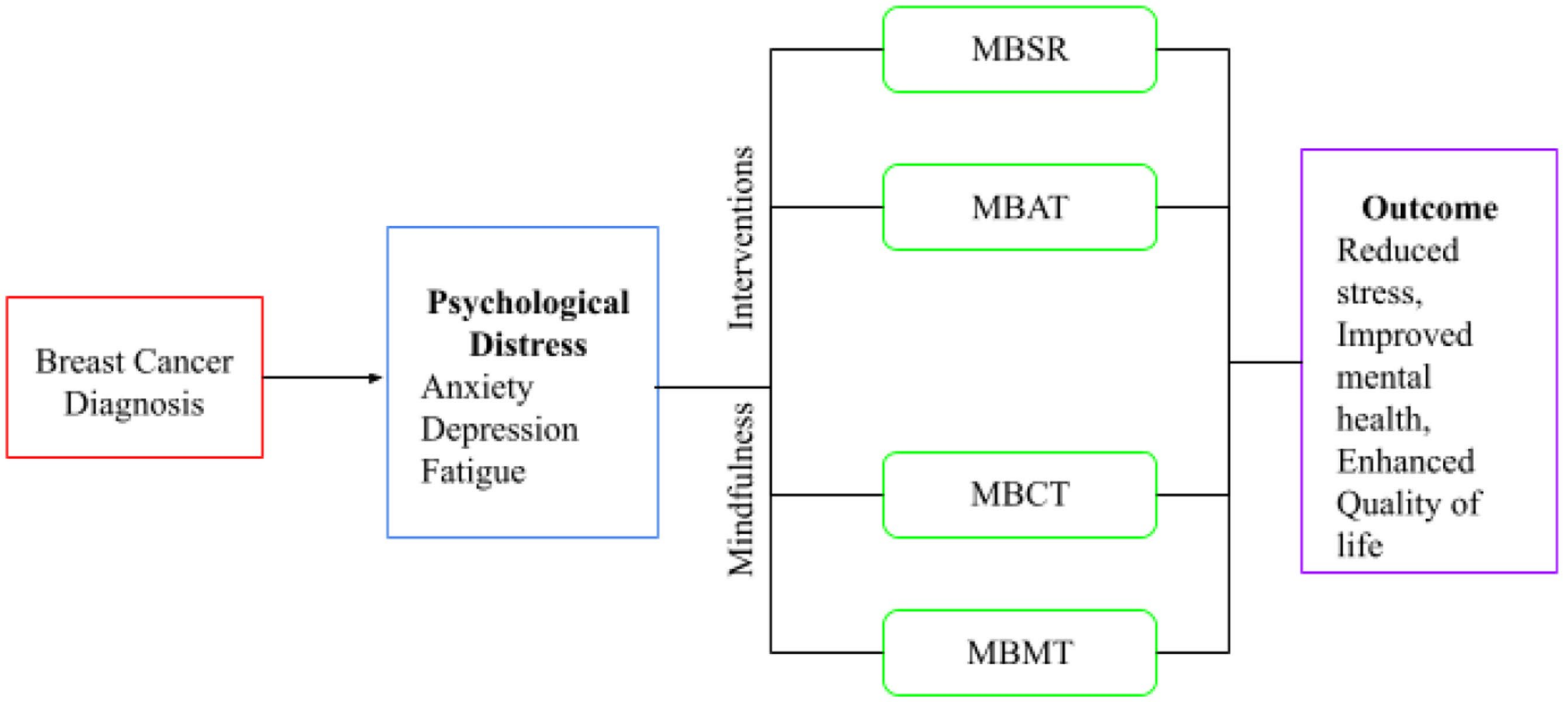
The diagram represents a conceptual framework illustrating the impact of Mindfulness practices on persons diagnosed with breast cancer QoL and mental health.

Across the 20 studies, sample sizes ranged from 30 to 1,200 participants, with most studies conducted in clinical or rehabilitation settings. Commonly used measures included the Hospital Anxiety and Depression Scale (HADS), Perceived Stress Scale (PSS), Functional Assessment of Cancer Therapy-Breast (FACT-B). Table 1 summarizes studies that demonstrated statistically significant positive effects of MBIs on psychological well-being and QoL among breast cancer patients and survivors. Collectively, these studies suggest that MBIs are effective in reducing psychological distress and enhancing mental health outcomes, contributing to improved emotional resilience, reduced symptom burden, and better overall adjustment to illness (Figure 2).

**Table 1:** Mindfulness/MBIs showed significant positive impact on mental health and/or QoL in breast cancer patients.

Author and year	Sample (*N*) and population characteristics	Method–design	Intervention	Control group	Timing (during/after treatment)	Measures
Yan et al. (2025)	*N* = 14 RCTs pooled; breast cancer patients	Meta-analysis of RCTs	Mindfulness therapy (varied formats)	Standard/usual care across trials	During and after treatment	HADS, Beck Depression Inventory (BDI), QoL measures
Wang et al. (2024)	Breast cancer patients (pooled RCTs)	Systematic review and meta-analysis	MBSR	Standard/usual care	During and after treatment	QoL (FACT-B), anxiety, depression
Naskar et al. (2024)	*N* = 80, breast cancer patients receiving chemotherapy	RCT	Mindfulness-based intervention (8 weeks)	Usual care	During treatment	PSS, QoL scales
Wang et al. (2022)	*N* = 102, breast cancer survivors	RCT	Internet-based MBCR (iMBCR, 6 weeks)	Waitlist	After treatment	EORTC QLQ-C30, HADS
McCloy et al. (2022)	*N* = 1,200 women with cancer (subgroup breast cancer)	Systematic review and meta-analysis of RCTs	MBIs (MBSR, MBCT)	Standard/usual care in included studies	During and after treatment	Fatigue, anxiety, depression, QoL scales
Wu et al. (2022)	*N* = 12 RCTs; breast cancer patients	Systematic review and meta-analysis	MBSR	Standard/usual care	During and after treatment	Anxiety, depression, QoL
Liu et al. (2022)	*N* = 150, breast cancer patients	RCT	MBSR (8 weeks)	Usual care	During treatment	PSQI, HADS, FACT-B
Joshi et al. (2021)	*N* = 60, breast cancer patients on chemotherapy	Mixed-methods quasi-experimental	MBAT (8 sessions)	No control	During treatment	Distress Thermometer, FACIT-Sp
Kang et al. (2021)	*N* = 48, breast cancer survivors	RCT	Internet-delivered MBSR (8 weeks)	Usual care	During treatment	Patient Health Questionnaire (PHQ-9), Pittsburgh Sleep Quality Index (PSQI), Generalized Anxiety Disorder 7 (GAD 7)
Park et al. (2020)	*N* = 135, Persons diagnosed with breast cancer	RCT	MBCT (8 weeks)	Usual care	During treatment	HADS, Fear of Recurrence Inventory, FACIT
Zhang et al. (2019)	*N* = 12 RCTs; women with breast cancer	Systematic review and meta-analysis	MBIs (MBSR, MBCT)	Standard/usual care in included studies	During and after treatment	QoL measures, psychological distress
Metin et al. (2019)	*N* = 60 breast cancer patients undergoing chemotherapy.	RCT	MBSR (12 sessions)	Progressive muscle relaxation (12 sessions)	During active treatment.	Fatigue Symptom Inventory (FSI), COPE Inventory, EORTC QLQ-C30
Jalambadani and Borji (2019)	*N* = 40 women diagnosed with breast cancer	Quasi-experimental, pre–post with control	MBAT (12 sessions)	Waitlist	Post treatment	World Health Organization Quality of Life-BREF (WHOQOL-BREF)
Pouy et al. (2018)	*N* = 30 breast cancer survivors.	Experimental, RCT	MBSR (8 sessions)	Routine follow-up	Survivorship phase (after treatment)	General Health Questionnaire (GHQ-28) and WHOQOL-BREF
Reich et al. (2017)	*N* = 322 breast cancer survivors	RCT	MBSR (8-week program)	Wait-list control	After treatment (survivors)	QoL (FACT-B), inflammatory cytokines
Lengacher et al. (2016)	*N* = 322, breast cancer survivors	RCT	MBSR (BC) (6 weeks)	Usual care	After treatment	Center for Epidemiologic Studies Depression Scale (CES-D), STAI, FACT-B
Lesiuk (2015)	*N* = 20, breast cancer patients receiving adjuvant chemotherapy	Pilot, small sample	MBMT (4 sessions)	No control	During treatment	POMS, Attention tests

Overall, the majority of studies demonstrated substantial reductions in anxiety, depression, fatigue, and stress levels, as well as notable improvements in QoL, emotional well-being, and coping mechanisms. However, not all interventions yielded uniform results. In contrast, Table 2 presents studies that did not find statistically significant improvements in psychological or quality-of-life outcomes following MBIs. Although these studies reported subjective improvements such as enhanced emotional awareness, greater coping ability, or reduced perceived stress, these outcomes did not reach statistical significance when compared to control groups.

**Table 2:** Mindfulness/MBIs did not lead to uniform significant effects across outcomes.

Author and year	Sample (*N*) and population	Method–design	Intervention	Control group	Timing (during/after treatment)	Measures
Komariah et al. (2025)	*N* = 104 breast cancer survivors	RCT	Remote-based mindfulness intervention	Usual care	After treatment	Sleep, fatigue, pain, physical function
Chang et al. (2022)	*N* = 91, breast cancer patients	RCT	MBSR (8 weeks)	Waitlist	During treatment	Female Sexual Function Index (FSFI), HADS
Shergill et al. (2022)	*N* = 85 breast cancer survivors with chronic neuropathic pain	RCT	MBSR (8 weeks)	Standard care	After treatment	Pain intensity, QoL, depression

The comparative analysis of the included studies reveals that several factors influenced the effectiveness of MBIs among breast cancer patients. Quality and duration emerged as critical determinants of success. Studies with structured, instructor-led programs lasting 8–10 weeks (e.g., Naskar et al., 2024; Yan et al., 2025; Lengacher et al., 2016) demonstrated significantly greater improvements in anxiety, depression, and QoL compared to those with brief or self-guided interventions. Modality of training also played a key role: in-person or hybrid formats showed more consistent results than exclusively online interventions, which were often limited by lower adherence and engagement (Komariah et al., 2025). Regarding inclusion criteria, trials that enrolled participants with moderate-to-severe baseline psychological distress (e.g., Joshi et al., 2021; Park et al., 2020) reported more substantial symptom reduction, whereas those with heterogeneous or asymptomatic samples often yielded non-significant findings.

Monitoring and outcome assessment varied widely across studies, influencing statistical significance. Studies employing validated multi-dimensional instruments such as HADS, PSS, FACT-B, and MAAS tended to report stronger effects, while those relying on single-domain or self-developed measures (e.g., Metin et al., 2019) showed inconsistent outcomes. The timing of the intervention relative to the cancer trajectory was another moderating factor. Interventions delivered during active treatment were more effective in reducing distress and fatigue, whereas those implemented post-treatment mainly improved emotional regulation and long-term well-being (Reich et al., 2017; Zhang et al., 2019).

Overall, these findings suggest that clinicians can employ mindfulness most effectively when interventions are well-structured, longer in duration, guided by trained facilitators, and timed to coincide with periods of heightened psychological vulnerability—such as during chemotherapy or immediately after treatment completion. Tailoring MBIs to patient needs, ensuring fidelity to established protocols like MBSR or MBCT, and integrating ongoing monitoring may maximize therapeutic outcomes and sustainability of benefits in breast cancer care.

## 4 Discussion

This systematic review reports evidence of 20 articles containing data related to the outcome of mindfulness on QoL and mental health in persons diagnosed with breast cancer. These studies provide compelling evidence that MBIs such as MBSR, MBAT, MBCT and MBMT can have a positive impact on various aspects of psychological and physical well-being among persons diagnosed with breast cancer undergoing treatment. These articles were published between 2015 and 2025. Despite the diversity of studies in the type of mindfulness practice, duration of intervention, study design, and assessment tools, 17 articles reported improvement in overall well-being.

The framework that shapes this review shows how mindfulness-based therapies connect with the mental health and overall well-being of breast cancer patients. Based on mindfulness ideas and ways to handle stress, it suggests that these therapies help people focus on the present, accept things without judgment, and manage their emotions better. This helps lessen negative behaviors like overthinking, worrying too much, and being overly stressed while improving healthy coping skills and strength.

Additionally, different factors like the cancer’s stage, when the therapy takes place (either during or after treatment), how long it lasts, and the way it is delivered (either face-to-face or online) can change how strong these connections are. This model helps explain why some therapies had strong results while others did not, which is important for finding the best mindfulness methods for helping with breast cancer treatment.

### 4.1 Ability of mindfulness interventions

#### 4.1.1 Stress reduction

Mindfulness interventions are particularly effective in reducing stress. The core principle of mindfulness involves focusing on the present moment and observing thoughts, feelings, and bodily sensations without judgment or emotional reactivity. This process helps individuals develop a greater sense of awareness and control over their stress responses. Studies have demonstrated that mindfulness can reduce both physiological and psychological stress in patients with chronic illnesses, such as breast cancer (Pouy et al., 2018; Naskar et al., 2024). These interventions help lower cortisol levels, a key stress hormone, and mitigate the body’s fight-or-flight response to stress, ultimately leading to improved emotional and physical well-being (Pascoe et al., 2017). Mindfulness techniques such as deep breathing, body scans, and meditation encourage relaxation and reduce symptoms of anxiety and tension, which are often exacerbated by cancer treatments and the fear of recurrence (Lengacher et al., 2009).

#### 4.1.2 Emotional regulation

Emotional regulation is the ability to manage and respond to emotional experiences in a healthy, adaptive way (Resilient Minds Psychology, 2024). Mindfulness interventions play a crucial role in enhancing emotional regulation by teaching individuals to observe and accept their emotions without becoming overwhelmed or acting impulsively. In the context of breast cancer, mindfulness practices can help patients process complex feelings of fear, sadness, anger, and grief that often accompany diagnosis and treatment. Mindfulness fosters a non-judgmental acceptance of emotions, which allows patients to experience and release negative emotions without becoming consumed by them (Park et al., 2020). This emotional regulation process helps to lower the likelihood of emotional reactivity, which can lead to further psychological distress, and supports individuals in navigating the emotional challenges that come with living with cancer. Through emotional awareness and acceptance, mindfulness enables patients to build resilience against mood swings and emotional distress, which is vital for improving QoL during cancer treatment and recovery.

#### 4.1.3 Improved coping mechanisms

One of the primary benefits of mindfulness interventions is the enhancement of coping mechanisms. Coping refers to the strategies individuals use to manage stress and adversity. Mindfulness helps individuals develop adaptive coping strategies by promoting awareness, acceptance, and focused attention, which empowers them to handle difficult situations with greater calmness and clarity. For persons diagnosed with breast cancer, this means learning to cope with the physical challenges of treatment, such as fatigue and pain, as well as the emotional and psychological burdens associated with their diagnosis (Carlson et al., 2021). Mindfulness practices help individuals reframe negative thought patterns, reduce rumination, and cultivate a greater sense of self-compassion, which improves their ability to adapt to ongoing stressors. By fostering greater acceptance of the present moment, mindfulness interventions help patients disengage from harmful thought cycles that might exacerbate feelings of helplessness or anxiety. As a result, patients can better manage the psychological burden of their illness and maintain a more positive and active outlook on life.

### 4.2 Motivation of mindfulness interventions

#### 4.2.1 Empowerment

One of the key motivational aspects of mindfulness interventions is the empowerment it provides to individuals. Empowerment in this context refers to the ability of patients to take control of their emotional, physical, and mental health by developing a sense of agency and mastery over their experiences. Mindfulness practices promote a sense of self-efficacy—individuals feel that they can influence their responses to the challenges they face, rather than simply being passive recipients of external stressors or emotional turmoil. Studies have shown that MBIs help persons diagnosed with breast cancer feel more in control of their psychological state and stress responses (Pouy et al., 2018; Park et al., 2020). Through mindfulness, individuals become more aware of their internal states, which allows them to recognize and address challenges proactively, rather than feeling overwhelmed. This sense of empowerment can foster a greater commitment to self-care practices and encourage patients to actively engage in their treatment and recovery processes.

#### 4.2.2 Positive self-perception

Mindfulness interventions also contribute to the development of a more positive self-perception. This is achieved by helping individuals cultivate self-compassion, non-judgment, and acceptance of themselves, which in turn promotes a healthier self-image. In the case of persons diagnosed with breast cancer, the psychological burden of diagnosis and treatment often includes feelings of inadequacy or a negative body image. Mindfulness practices enable individuals to acknowledge these feelings without judgment, allowing them to accept their experiences and focus on their strengths rather than their perceived flaws (Carlson et al., 2021). This shift in perspective is crucial for improving self-esteem and self-worth, especially when facing the physical and emotional challenges of cancer treatment. By fostering a more positive self-view, mindfulness can motivate individuals to engage in life with more hope, resilience, and self-acceptance, which supports their overall well-being and mental health.

#### 4.2.3 Improved quality of life

Mindfulness interventions are also highly motivating because they contribute to improved QoL, particularly in terms of emotional and physical well-being. A key motivator for patients engaging in mindfulness practices is the potential to alleviate the discomfort and distress associated with illness. Mindfulness improves emotional well-being by promoting emotional regulation and reducing negative mental states, such as anxiety, depression, and stress. Research has shown that mindfulness can lead to increased life satisfaction, better mood regulation, and greater overall contentment with life (Naskar et al., 2024). In persons diagnosed with breast cancer, mindfulness can reduce fatigue, enhance coping skills, and improve both physical and emotional QoL, which motivates individuals to continue engaging in the intervention (Lesiuk, 2015). As patients experience tangible improvements in their mental and physical health, their motivation to continue practicing mindfulness grows, creating a positive feedback loop that enhances their QoL throughout their cancer journey.

### 4.3 Opportunity of mindfulness interventions

#### 4.3.1 Accessibility

Mindfulness interventions have become more accessible due to their adaptability in delivery methods, such as online programs, mobile applications, and in-person workshops. For example, a study by Tighe et al. (2017) examined the use of an online MBSR program for cancer patients. The results indicated that patients who participated in an online version of MBSR experienced significant reductions in psychological distress and improvements in overall well-being. This form of delivery allows patients who may have mobility issues or live in rural areas with limited access to traditional therapies to participate in therapeutic programs from the comfort of their homes. Similarly, mobile apps, like those used in studies by Goyal et al. (2014), have been shown to be an effective means of making mindfulness practices available to a broader population. These methods make mindfulness interventions accessible to a wider range of individuals, especially those undergoing cancer treatment, where attending in-person sessions may be difficult due to the demands of treatment or physical limitations.

#### 4.3.2 Group support

Mindfulness interventions that are offered in group settings have been shown to provide significant psychological benefits through the social support they offer. Group-based mindfulness programs create a sense of community among participants who share similar experiences, such as undergoing cancer treatment. Raab et al. (2014) conducted a study where patients with breast cancer participated in a group-based MBSR program. The results showed not only reductions in stress but also an enhanced sense of social connection among participants. This collective experience fosters emotional healing as individuals share their challenges and coping strategies, leading to improved psychological resilience. The sense of shared experience and mutual support that comes from engaging in a group-based intervention is especially valuable for persons diagnosed with breast cancer, who often experience feelings of isolation and anxiety. Group mindfulness programs help patients feel less alone in their struggles, thereby fostering a supportive environment where they can develop healthier coping mechanisms together.

#### 4.3.3 Integration with medical care

The integration of MBIs with traditional medical treatments is another promising opportunity for improving patient outcomes. Mindfulness interventions can serve as complementary therapies alongside chemotherapy, radiation, and other medical treatments, addressing both the physical and emotional components of the cancer experience. Feldman et al. (2017) conducted a study where persons diagnosed with breast cancer underwent an MBSR program while receiving medical treatment. The results demonstrated significant reductions in anxiety and depressive symptoms, as well as improvements in the patients’ QoL. Integrating mindfulness into medical care allows for a more holistic approach to treating cancer, addressing the physical side effects of treatment (such as fatigue, nausea, and pain) while simultaneously providing psychological relief. Furthermore, healthcare providers can offer mindfulness practices to patients as part of their treatment regimen, providing an additional coping mechanism that can reduce treatment-related distress and improve overall outcomes.

The results of this review show that mindfulness programs, like Mindfulness-Based Stress Reduction, Mindfulness-Based Cognitive Therapy, and Mindfulness-Based Art Therapy, have a strong positive effect on mental health and overall QoL for people with breast cancer. Out of the 20 studies included, most of them (17) found real improvements in anxiety, depression, stress, tiredness, and emotional health, while a few (three) did not show any significant benefits. These results highlight how helpful mindfulness can be alongside regular cancer treatments, especially for the mental challenges that often come with a breast cancer diagnosis and treatment.

Mindfulness programs seem to help people manage their emotions better, think more flexibly, and cope in constructive ways, making it easier to handle the many stresses of having cancer. Because these methods are safe, affordable, and can be easily shared, they could be important parts of cancer care that combine different approaches. Adding mindfulness programs to supportive care services or recovery plans could greatly improve mental well-being and help create a more complete form of patient-focused care. Additionally, new information about online or mixed formats of mindfulness programs indicates that they can still be effective while being easily accessible, which helps reach individuals who may not have easy access to these supports or who live far away.

Despite these positive results, we need to keep in mind some problems with how we interpret the findings. Even though looking at a broader range of studies helped us find a lot more options, only 20 of those studies were actually picked for a closer look. This smaller selection, though backed by solid evaluation methods, might restrict the overall application of the findings and could introduce a bias in what was chosen. There were significant differences in how the studies were set up, the treatments that were provided, and the backgrounds of the participants. Some studies did not sort participants by their cancer stage (like early, later, or spreading), the type of treatment they received, or how long they had been living with the illness. These aspects could influence how effective mindfulness is for their mental well-being. Moreover, the treatments varied greatly in duration and intensity—from short 4-week programs to longer ones that lasted eight or 12 weeks—making it difficult to compare results across different studies and to investigate how the amount of treatment could impact the outcomes. Another issue is that the way measurements were taken was not uniform. Different studies used a range of recognized methods to assess anxiety, depression, stress, and QoL, like the Hospital Anxiety and Depression Scale, Perceived Stress Scale, Functional Assessment of Cancer Therapy—Breast, and the European Organization for Research and Treatment of Cancer Quality of Life Questionnaire. While this variety shows a comprehensive approach to evaluating these matters, it complicates the reliability of the overall results and makes comparing the findings accurately more challenging. Although including studies published in other languages helped lessen biases related to language differences, most of the studies came from Western or East Asian regions, with very few originating from lower- or middle-income countries. This makes it tough to generalize the findings to other environments and highlights the need for research that considers cultural variations, especially in countries like India, where psychosocial oncology is beginning to develop. Additionally, not having a proper evaluation of bias risks and failing to register studies in well-known databases like PROSPERO or OSF are other issues in the research methods. These gaps can make the research process less transparent and increase the likelihood of selective reporting. Future systematic reviews should utilize standard tools for quality assessment and create preregistration protocols to enhance the quality and reliability of the research.

A comparison of the findings shows that how well mindfulness programs work relies on several connected factors. These include how well the programs are followed, how long they last, the skills of the instructors, how involved the participants are, and how they fit with the patients’ treatment paths. Research that shows no significant results often used short interventions, did not have strong follow-up plans, or included a mix of different people without proper control groups. On the other hand, programs that followed set guidelines like MBSR or MBCT and ran for at least 8 weeks regularly reported good results. Future studies should focus on using the same intervention guidelines, consistently applying trusted outcome measures, and designing long-term studies to understand how lasting the effects are. Also, more effort should be put into figuring out the best time to deliver these interventions during the cancer care journey—whether it is while patients are getting treatment, during recovery, or in the long-term after surviving. From a medical perspective, adding MBIs into a team approach to cancer care could be a great way to meet the emotional and social needs of breast cancer patients, helping them have a better QoL and become stronger throughout their cancer experiences.

## 5 Conclusion

This review shows that mindfulness practices can be a helpful addition to standard breast cancer treatments. They offer clear benefits for mental health, managing emotions, and improving overall QoL. By looking at 20 different studies, we see that mindfulness programs—especially ones like MBSR and MBCT—are useful, easy to access, and can be adjusted for different settings and cultures. However, there are differences in how the studies were organized, how long the mindfulness sessions lasted, and the types of patients involved, which points to the need for more consistency and long-term studies. Future research should aim to find the best ways to deliver these programs, figure out what makes treatments work better for some people, and create versions that are suitable for various cultural groups. Adding mindfulness to cancer care shows a lot of potential for improving care that focuses on patients as a whole and helps women with breast cancer feel better emotionally and socially.

## Data Availability

Data will be available on request from corresponding author.
